# Impulsivity and aggression as risk factors for internet gaming disorder among university students

**DOI:** 10.1038/s41598-024-53807-5

**Published:** 2024-02-14

**Authors:** Mohammad Ahmed Hammad, Hend Faye AL-shahrani

**Affiliations:** 1 Faculty of Education, Najran University, Najran, Kingdom of Saudi Arabia; 2 Department of Social Work, College of Humanities and Social Sciences, Princess Nourah Bint Abdulrahman University, Riyadh, Kingdom of Saudi Arabia

**Keywords:** Internet gaming disorder, Impulsivity, Aggression, Young adults, Psychology, Human behaviour

## Abstract

Internet gaming addiction is a global problem, especially among young individuals. Exhibiting characteristics similar to other addictions, Internet Gaming Disorder (IGD) is linked to adverse mental health outcomes. Identified as risk factors for dependence behaviors, the association of impulsivity and aggression with IGD is relatively under-researched in the student population. The present sample of 350 university students (M_age_ = 21.30 years, SD_age_ = 4.96 years) from Najran university in Saudi Arabia completed an online questionnaire that included the Internet Gaming Disorder Scale–Short-Form (IGDS9-SF), the Buss–Perry Aggression Questionnaire-Short Form, and the Barratt Impulsiveness Scale (BIS-15). Results indicated that impulsivity and aggression were positively associated with IGD severity and both personality traits explained 34.6% of the variance in IGD scores. Further bivariate analyses suggested that individuals spending 7 or more hours on internet gaming were more likely to exhibit high impulsivity and aggression, and had a relatively higher severity of IGD. These results suggest that individuals with these personality traits may be more vulnerable to developing an addiction to internet gaming. These findings need to be confirmed in future more robust studies; however, this exploratory study provides insights for potential programs to prevent IGD among young individuals.

## Introduction

With its use ranging from information seeking, expression of opinions, entertainment, social networking and many other such functions, the Internet has become an integral part of our lives. As of 2022, about 66% of the global population was reported to use the internet^1^ Considering Saudi Arabia alone, the internet penetration rate has increased from about 87% in 2018 to 98% in 2023^2^. This exponential growth in internet accessibility and use has raised justified concerns about possible addiction^3^. Problematic internet use has been found to be as detrimental as any other addictive behavior such as overeating, alcoholism, drug use, and other forms of substance dependence^4^. However, newer research in this field has explored the specific effects of online gaming, especially since DSM-5 identified it as a disorder warranting further research^5^. Classified as a non-substance addiction^6^, online gaming disorder or internet gaming disorder (IGD), is defined as the continuous and frequent use of online games that causes clinically significant impairment or distress^5^. While its real prevalence is unclear, primarily due to the lack of consensus in its characteristics, definition, and diagnosis^7^, a recent meta-analysis reported an overall prevalence of 0.7–27.5% based on 13 longitudinal studies and 37 cross-sectional studies^8^. Another meta-analysis of 155 studies from 33 countries reported a pooled prevalence of 8.8% among adolescents and 10.4% among young adults^9^.

The increasing popularity of online gaming and concurrent global investment in the online gaming industry point to the emergence of a very real public health concern. Furthermore, with high prevalence of IGD among adolescents and young adults, there has been increasing research focus on the negative effects of IGD on physical and mental health outcomes^10^. Higher levels of IGD have been found to be associated with several problems such as impulsivity, aggression, low social acceptance, low academic achievement, disturbed parent–child relationships, anxiety and depression, loneliness, and emotional distress^9–16^. As such, several researchers have confirmed that the psychosocial and behavioral characteristics observed in IGD are similar to those in substance use disorder.

For instance, building on research confirming the role of impulsivity in substance use disorder, several researchers have identified it as a contributing factor in IGD^6,17–20^. An fMRI study that compared 17 adolescents with and without internet gaming addiction found that the former group exhibited higher scores on the Barratt Impulsivity Scale 11 and showed impaired functioning in the brain regions responsible for impulse control^17^. These results support the association between impulsivity and IGD. Loss of control in online games is a key criterion for diagnosing IGD^21^, possibly because extreme impulsivity can cause individuals to succumb to the rewarding effects of gaming and contribute to an increase in IGD. Similarly, a questionnaire-based longitudinal study reported that self-contained impulsivity was a risk factor for IGD among adolescents^11^. Aggression is another psychological symptom observed in substance use disorders that has also found to be associated with IGD^22–25^, For instance, in an empirical study, Barlett et al.^26^ found that an increase in violent game participation led to a concurrent increase in aggression measures. In another prospective study among adolescents, aggression was found to be a strong predictor of internet addiction^27^. It is assumed that individuals play online games as a means of releasing their aggressive impulses and seeking sensation to alleviate boredom^6^.

In light of the above literature, it is clear that with the growing popularity of online games, especially among young individuals, there are concerns about their excessive use^28^. As concerns about IGD continue to increase, mental health practitioners and public health policy makers need to gain a better understanding of the various factors influencing IGD in this population. Therefore, the present study aimed to examine the association of impulsivity and aggression with IGD among young individuals.

### The present study

Research on IGD is relatively new, more so among young individuals. The impacts of disordered internet gaming in this population includes poor mental health outcomes (e.g., depression and anxiety, stress, emotional distress)^29^, as well as negative effects on academic performance, general health and well-being (including sleep patterns), as well as social participation (such as that in leisure activities)^30,31^. This population is the future generation that will contribute to the labor market and overall social, economic, and political development. With their increased vulnerability to IGD owing to substantial exposure to the internet and mobile devices, it is important to conduct more research on this population.

Existing research on IGD in university students has mainly focused on prevalence and related socio-demographic factors. Within those, owing to differences in research design, diagnostic criteria and tools used, population differences, etc., most studies report varying incidences. With two studies reporting an prevalence of about 5% among Mexican first-year university students^32^ and Iraqi undergraduate students^33^, most others reported an prevalence ranging from 9 to 16% among Egyptian university students^34^, medical and dental students in India^35^, male college students in Nanchong, China^36^, medical students in Indonesia^37^, and US college students^29^. Recent studies in Saudi Arabia reported a prevalence of 17–30% among adolescents^38,39^ and the general population^7^. Studies more specific to Saudi university students reported rates of 10.1%^40^ and 21.5%^41^, and that of 8.8% among medical students^42^. While most of these studies have focused on the diagnosis and prevalence of IGD, there is a paucity of research on associated risk factors among Saudi university students. As mentioned before, the internet penetration rate in Saudi Arabia is 98%^2^. Therefore, we are looking at a potential public health crisis waiting to happen. As such, focusing on university students was considered of prime importance in the present study. With the purpose of identifying probable risk factors for IGD in this population, this exploratory study aimed to examine the association of impulsivity and aggression with IGD among Najran university students in Saudi Arabia.

## Materials and methods

### Sample

The present cross-sectional, exploratory study, the sample was selected through simple random sampling technique from the aggregate of students enrolled in Najran University, Saudi Arabia. The required sample size was determined using G*Power 3.1^43^. Inputting a 0.05 α error probability and 0.90 power, the recommended sample size for an ANOVA with multiple predictors was 338. Therefore, allowing for response rate adjustments, the study invite was sent to 382 students randomly via social media groups. Of them, excluding incomplete responses and non-consent, the final sample comprised 350 students, which was still over the recommended sample size for the present study. To be eligible for the present study, the students had to be aged over 18 years, be currently enrolled in a 4-year degree program at Najran University (in the 2022–2023 academic year), be capable of communicating in Arabic or English, and consent to participate in the study.

### Data collection

#### Procedure

**Data collection:** Data were collected via an online survey from January to March 2023. Randomly selected potential participants from the 4-year degree courses at Najran University were sent a study invite that contained an information package and the study questionnaire (Google Forms). The information package explained the purpose and procedures of the study and the participants’ rights (anonymity and confidentiality, voluntary consent, non-maleficence, and freedom to withdraw consent at any point during the study). Prior to its commencement, all procedures performed in study involving human participants were in accordance with the ethical standards of the institutional and/or national research committee and with the 1964 Declaration of Helsinki and its later amendments or comparable ethical standards. Informed consent was obtained from all individual participants included in the study. In addition, the protocol for the study was approved by Research Ethics Committee at the University.

#### Instruments

##### Sociodemographic questionnaire

The questionnaire included questions on the participants’ gender, age, academic specialty and time spent playing games online.

##### Internet Gaming Disorder Scale–Short-Form (IGDS9-SF)

The IGDS9-SF^44^ is a 9-item screening tool to identify IGD based on the nine core criteria outlined in the DSM-5^5^. It assesses the severity of IGD based on the frequency of gaming activities in the past 12 months. The scale uses a 5-point Likert scale (1 = never to 5 = often), with total scores ranging from 9 to 45 points. Higher scores indicate a higher severity of gaming disorder. Furthermore, Qin et al.^45^ recommend a cut-off of 32 points to determine the presence or absence of gaming disorder. A recent meta-analysis of 21 studies examining the psychometric properties of 15 language versions of the IGDS9-SF reported good reliability and validity over several metrics, and also confirmed its cross-cultural validity^46^. In the present study, the IGDS9-SF had high reliability with an internal consistency coefficient (Cronbach's α) of 0.92, as suggested by several previous studies e.g.^3,12, 18, 44^.

##### Barratt Impulsiveness Scale (BIS-15)

The BIS-15^47^ is an abbreviated 15-item version of the original 30-item BIS-11^48^, which assesses impulsive personality traits. BIS-15 matched the original scale’s three-factor structure, comprising 3 sub-scales, viz., non-planning impulsivity, motor impulsivity, and attentional impulsivity. Response options are presented on a 4-point Likert scale (rare/never = 1, sometimes = 2, often = 3, almost always/always = 4), with total scores ranging from 15 to 60 points, Higher scores indicating more impulsiveness. The BIS-15 has exhibited high internal consistency in several previous studies e.g.^47,49^. In the current study too, the BIS-15 showed high internal consistency, with a Cronbach's α of 0.81.

#### The Buss–Perry Aggression Questionnaire (BPAQ-SF)

The BPAQ-SF^50^, a 12-item revised version of the original 29-item BPAQ^51^. The BPAQ-SF assesses aggression across the four dimensions of physical aggression (3 items), verbal aggression (3 items), anger (3 items), and hostility (3 items). Items are rated on a 5-point Likert scale (never to always), with total scores ranging from 12 to 60 points, Higher scores indicate higher levels of aggression. Several previous studies have reported internal consistency e.g.^50,52, 53^, similar to that observed in the present study (Cronbach's α = 0.86).

### Data analysis

Statistical analyzes were performed using SPSS version 21. As the 3 assessment tools were translated into Arabic for this study, their internal consistency was assessed using Cronbach's α coefficients. In addition, appropriate descriptive statistics were computed (*M* and *SD* or *f* and %) as a first step in the analyses. For bivariate and multivariate comparisons, *t*-test, Chi-square, and ANOVA were used as appropriate, after testing for normality and homoscedasticity. Finally, correlation and regression analyses were used to examine the association of IGD with impulsivity and aggression, while controlling for relevant demographic variables.

### Ethical approval

All procedures performed in studies involving human participants were in accordance with the ethical standards of the institutional and/or national research committee and with the 1964 Declaration of Helsinki and its later amendments or comparable ethical standards.

### Informed consent

Informed consent was obtained from all individual participants included in the study. In addition, the protocol for the study was approved by Research Ethics Committee at the University.

## Results

### Understanding IGD in Saudi University students

The present sample of 350 students from Najran university in Saudi Arabia, Table 1 presents the characteristics of the final sample. The mean IGD severity score was 27.23 (SD = 6.087; range 12–40), with the tool’s possible score ranging from 9–45 points. Further, using the 32-point cutoff suggested by Qin et al.^45^ to identify disordered gamers, 21.71% of the sample scored 33 points or over. There were no statistically significant group differences in mean IGD scores based on gender, academic specialty, and age. However, a one-way ANOVA revealed that the mean IGD score differed significantly when considering weekly hours spent on online gaming (*F* = 84.68(3, 346), *p* = 0.001).

**Table 1:** Sample characteristics (*N* = 350).

Variables	Categories	*f* (%)
Age	–	21.30 (4.96) years
Gender	Male	186 (53.1%)
	Female	164 (46.9%)
Academic specialty	Humanities and social sciences	222 (63.4%)
	Applied sciences	128 (36.6)
Weekly gaming duration	≤ 1 h	4 (1.1%)
	2–6 h	165 (47.1%)
	7–19 h	95 (27.1%)
	≥ 20 h	86 (24.6%)

Post-hoc analyses revealed that those who played online games for 7–19 h per week, and ≥ 20 h per week had significantly higher IGD scores than the other two groups, and the latter group had the highest score across all other groups of weekly hours spent on gaming (all *p*s < 0.001).

Furthermore, a Chi-squared test comparing belongingness to the IGD or non-IGD group (according to the 32-point cutoff) based on weekly gaming duration revealed significant differences (χ^2^ = 149.02(3), *p* = 0.001). Specifically, those who spent ≥ 20 h per week were more likely to have IGD (Table 2). Furthermore, when examining the groups’ means (Table 3) it was evident that the mean IGD score of the 7–19 h per week group was closer to the 32-point cutoff for disordered gaming. The relevance of these findings have been examined further in the Discussion section. However, as weekly gaming duration emerged as an important intervening factor, it was included as a control variable in the later regression analysis.

**Table 2:** Distribution of IGD and non-IGD groups based on weekly gaming duration (*N* = 350).

Gaming duration (hours/week)	IGD group *f* (%)	Non-IGD group *f* (%)
≤ 1	0	4 (1.14%)
2–6	2 (0.57%)	163 (46.57%)
7–19	16 (4.57%)	79 (22.57%)
≥ 20	58 (16.57%)	28 (8%)

**Table 3:** Results of the one-way ANOVA on IGD scores across weekly duration of online gaming (*N* = 350).

Gaming duration groups (hours/week)	IGD Score Mean (*SD*)	Gaming duration comparison groups (hours/week)	Mean difference	Std. error
≤ 1	17.75 (4.5)	2–6	− 5.87 (n.s.)	2.342
		7–19	− 11.03*	2.363
		≥ 20	− 15.11*	2.368
2–6	23.62 (4.92)	≤ 1	5.87 (n.s.)	2.342
		7–19	− 5.15*	0.596
		≥ 20	− 9.24*	0.616
7–19	28.78 (4.52)	≤ 1	11.03*	2.363
		2–6	5.15*	0.596
		≥ 20	− 4.08*	0.689
≥ 20	32.86 (4.23)	≤ 1	15.11*	2.368
		2–6	9.24*	0.616
		7–19	4.08*	0.689

*p < 0.001.

### Association of impulsivity and aggression with IGD scores

The overall mean score for the entire sample was 35.60 (*SD* = 7.16) points on the impulsivity scale (BIS-15), with a possible score ranging from 15–60 points. The mean score on the aggression scale (BAPQ-SF) was 28.85 (*SD* = 6.88) points, with a possible score ranging from 12–60 points. A *t*-test was conducted to examine IGD/non-IGD group differences in impulsivity and aggression scores. As evident from Table 4, those in the IGD group tended to have higher scores on the impulsivity and aggression scales.

**Table 4:** Comparing IGD and non-IGD groups on impulsivity and aggression scores (*N* = 350).

	Group	*f*	*M*	*SD*	*t* (df)	*p*
Impulsivity	IGD	76	41.25	5.185	8.537 (348)	0.001
	Non-IGD	274	34.03	6.842		
Aggression	IGD	76	37.00	3.171	14.929 (348)	0.001
	Non-IGD	274	26.58	5.844		

Theoretical range for the impulsivity and aggression scales was 15–60 and 12–60 points, respectively.

Further, a Pearson’s correlation test revealed a significant, moderately strong, positive correlation between IGD and impulsivity (*r* = 0.68, *p* = 0.001), and a significant, strong, positive correlation between IGD and aggression (*r* = 0.92, *p* = 0.001). These findings suggest that higher IGD scores were associated with higher impulsivity and aggression scores, and vice versa.

Finally, a step-wise hierarchical regression analysis was conducted while controlling for the effects of weekly gaming duration. Other demographic variables such as gender, academic specialty, and age were not included in the regression because the bivariate analyses did not reveal significant group differences in the present sample. Furthermore, for use in the regression model, a binary variable was created for weekly gaming duration using 7 h as a cutoff based on the ANOVA results presented in Table 3. Findings of the regression analysis revealed that, when controlling for weekly gaming duration, impulsivity and aggression were significant predictors of IGD scores, explaining 34.6% of its variance (R^2^ = 0.37, adjusted R^2^ = 0.346, *F*(3, 346) = 67.92, *p* = 0.001).

## Discussion

The present exploratory was the first of its kind to examine the possible role of impulsivity and aggression as risk factors for IGD among university students in Saudi Arabia. About 22% of the participants were found to have IGD that was severe enough to categorize them as disordered gamers as per the recommended 32-point cutoff. This prevalence rate is substantially higher than those reported in recent studies on university students in Egypt 9.3%^34^, Indian medical students 9%^35^, Saudi medical students 8.8%^42^, Saudi university students 10.1%^40^. However, it is very similar to the findings of Alsunni and Latif^41^, who reported an incidence of 21.5% among Saudi university students. It is important to note that, while Alsunni and Latif^41^ used the nine criteria outlined in the DSM-5, the tool used in the present study is also based on these criteria and has exhibited excellent psychometric properties^46^. Possibly, that may explain why these two studies show similar incidences.

The evident differences in prevalence rates among previous studies on university students stem from differences in sample and sample sizes, population, and most importantly, the diagnostic criteria and assessment tools used. Further, the 32-point cutoff recommended by Qin et al.^45^ to identify disordered gamers based on their IGDSF-9 scores is yet to be clinically validated on a cross-cultural sample. Nevertheless, they do acknowledge that the cutoff has enough reliability for research use. Indeed, future studies need to examine the sensitivity and specificity of this cutoff on large, culture-fair samples. Another implication of the higher prevalence in the present study as compared to the above-cited studies could be the timing of the study. It may be noteworthy that most of the studies reporting an prevalence of 8–10% were conducted before the COVID-19 pandemic. The effects of the pandemic and related social isolation are well established. As highlighted by Avena et al.^54^, as individuals did not have access to mental health support during this period, there was a sharp increase in substance use disorders during COVID-19. A concurrent increase in gaming behavior during social isolation was also observed among college students in India^55,56^, and among adolescents in SA^57^ and Korea^58^. The increase in gaming, increase in stressors related to the pandemic, and simultaneous lack of access to mental health services may explain the sharp increase in the prevalence of IGD post-pandemic. However, this is mere conjecture in the absence of comparative data. Notwithstanding the causes for different results, the present finding of over 20% prevalence of IGD is worrisome from a public health perspective. It highlights the need for more urgent research on this population to gain a holistic understanding of the risk and protective factors influencing IGD in young individuals.

Though it was not the main focus of the present study, one influencing factor incidentally observed in the results was weekly gaming duration. A one-way ANOVA comparing IGD means across gaming duration groups, concurrent post-hoc analyses, and a Chi-squared test comparing IGD-non-IGD group belongingness with gaming duration confirmed that those who played for over 20 h per week had higher scores on the IGD scale and they were more likely to fall in the disordered gamers’ group. Further, those who played for 7–19 h per week followed closely in terms of their mean IGD score, and an additional 17% of them fell in the disordered gamers’ group (derived from Table 2). These findings were not surprising given that several previous studies have linked gaming duration to higher IGD severity^7,59, 60^. In their meta-analysis of 155 studies on adolescents and young adults from 33 countries, Gao et al.^9^ also found time spent on playing as an important risk factor along with other psychosocial factors. This was corroborated by Ropovik et al.^10^ from their meta-analysis of 253 studies on IGD and its factors. Together, the present findings and those of previous studies and meta-analyses suggest that we need to be mindful of the amount of time young individuals spend on gaming, as it is a strong risk factor for developing IGD. Further, while the negative effects of gaming for > 20 h a week are quite evident^7^, the present results suggest that there still may be hope for the 7–19 h group because though they may be at risk of IGD, there is still hope for timely preventive intervention for most of them.

With reference to the core focus of this study, that is, the association between IGD symptoms, impulsivity, and aggression, results of the *t*-test, correlation test, and step-wise hierarchical regression together confirmed that higher IGD scores were associated with higher impulsivity and aggression scores. Both factors were separately correlated with IGD, and together they explained 34.6% of the variance in IGD scores after controlling for the effects of gaming duration. As explained in the introduction section, impulsivity has been identified as a significant factor in substance use disorders, and IGD has also been confirmed to share its characteristics with other addictive behaviors^6,17–19^. More specifically, impulsivity was identified as a significant risk factor for IGD in various samples^32^ and high impulsivity was associated with higher severity of IGD among Korean adults^61^ and Turkish university students^62^. Therefore, researchers suggest that IGD is primarily an impulse control disorder^63^. One possible explanations for most of these findings can be derived from neurobiological studies that found impaired attention and executive functions^14^, lower activity in the frontal lobe and amygdala (which are responsible for executive functions, and stress response and emotions, respectively)^64^, poor impulse control, emotional regulation, decision-making, and working memory^65^, resulting in loss of control over internet use. Another influencing mechanism may be the instant gratification derived from games, which is related to the response and reward system^17^, thereby strengthening pre-existing impulse control issues. This reward mechanism also entails the role of dopamine release during gaming, which has been considered similar to the dopamine surge cause during drug abuse^66^.

The present findings also revealed a strong association between aggression and IGD, which corroborates extant findings^6,23, 26, 27^. This may be because games provide a legal platform for the expression of aggression. Further, the anonymity of the online gaming environment^67^ and the opportunity to act out their fantasies, aggression, and anger without restrictions or judgement^23^ may be attractive for gamers. In most games, aggressive behavior is rewarded, which may convert aggression to goal-oriented behavior; thus, leading to a vicious circle that eventually causes an addiction to this form of outlet^27,47, 67^. The relationship between aggressive personality and preference for aggressive games is established^68^. Other studies have reported a significant positive association between IGD severity and physical aggression^67^. Similarly, Yu and Cho^69^ revealed that the group with IGD had the highest degree of physical aggression, anxiety and depression as compared to the non-IGD group. Together, the present findings add to the existing literature on IGD, especially among young individuals. However, the study has several limitations that need to be considered when interpreting and generalizing these findings.

First, it is difficult to generalize the results to other population groups because this study was conducted on a small sample of students from one university in Saudi Arabia, Therefore, the present probable risk factors data, as well as other findings are more specific to the population that may share characteristics with the present limited sample of Najran University students in Saudi Arabia. Further, the use of an online survey with self-administered questionnaires, and subjective reports of duration of online gaming introduce bias in the methodology, dataset, and findings derived from it. Finally, the cross-sectional design employed in this study does not allow for the determination of nature and direction of causality between variables. The independent variables explained about 35% of the variance in IGD scores, which is not small but suggests the presence of other influencing factors. Specifically, the role of family, peers, community, and sociocultural factors must be clarified. Future studies need to disentangle the individual and combined effect of these factors on impulsivity, aggression, and IGD among university students. Most of the methodological limitations mentioned above can be addressed by conducting future studies with a larger, more diverse samples, and through longitudinal and qualitative studies to establish causality and clarify underlying mechanisms of influence.

## Conclusions and implications

The main objective of this study was to determine the association of impulsivity and aggression with IGD among university students in Saudi Arabia. The results of the study showed that there is a positive correlation between IGD and impulsivity and aggression. That is, individuals who are impulsive and aggressive may be more vulnerable to developing an addiction to internet gaming. Furthermore, the relationship between IGD and impulsivity and aggression is moderated by several variables including weekly duration of online gaming. Despite its limitations, this study highlights the importance of monitoring the impulsivity and aggression of individuals who engage in internet gaming, as this may help prevent excessive gaming behavior from developing into an addiction. The present results also highlight the need to develop interventions and strategies to prevent IGD.

## Data Availability

The datasets used and/or analyzed during the current study are available from the corresponding author on reasonable request.
